# Health-related quality of life, neuropsychiatric symptoms and structural brain changes in clinically isolated syndrome

**DOI:** 10.1371/journal.pone.0200254

**Published:** 2018-07-06

**Authors:** Eva Hyncicova, Adam Kalina, Martin Vyhnalek, Tomas Nikolai, Lukas Martinkovic, Jiri Lisy, Jakub Hort, Eva Meluzinova, Jan Laczó

**Affiliations:** 1 Department of Neurology, Charles University, 2nd Faculty of Medicine, Motol University Hospital, Prague, Czech Republic; 2 Department of Radiology, Charles University, 2nd Faculty of Medicine, Motol University Hospital, Prague, Czech Republic; McLean Hospital, UNITED STATES

## Abstract

**Background:**

Neuropsychiatric symptoms and reduced health-related quality of life (HRQoL) are frequent in multiple sclerosis, where are associated with structural brain changes, but have been less studied in clinically isolated syndrome (CIS).

**Objective:**

To characterize HRQoL, neuropsychiatric symptoms (depressive symptoms, anxiety, apathy and fatigue), their interrelations and associations with structural brain changes in CIS.

**Methods:**

Patients with CIS (n = 67) and demographically matched healthy controls (n = 46) underwent neurological and psychological examinations including assessment of HRQoL, neuropsychiatric symptoms and cognitive functioning, and MRI brain scan with global, regional and lesion load volume measurement.

**Results:**

The CIS group had more, mostly mild, depressive symptoms and anxiety, and lower HRQoL physical and social subscores (p≤0.037). Neuropsychiatric symptoms were associated with most HRQoL subscores (β≤-0.34, p≤0.005). Cognitive functioning unlike clinical disability was associated with depressive symptoms and lower HRQoL emotional subscores (β≤-0.29, p≤0.019). Depressive symptoms and apathy were associated with right temporal, left insular and right occipital lesion load (ß≥0.29, p≤0.032). Anxiety was associated with lower white matter volume (ß = -0.25, p = 0.045).

**Conclusion:**

Mild depressive symptoms and anxiety with decreased HRQoL are present in patients with CIS. Neuropsychiatric symptoms contributing to decreased HRQoL are the result of structural brain changes and require complex therapeutic approach in patients with CIS.

## Introduction

Multiple sclerosis (MS) is a chronic demyelinating disease of the central nervous system associated with physical disability, cognitive dysfunction and increased frequency of neuropsychiatric symptoms including depressive symptoms, anxiety, apathy and fatigue that have a negative impact on health-related quality of life (HRQoL) [1, 2]. Associations of cognitive dysfunction, depressive symptoms, anxiety, apathy and fatigue with multiple components of HRQoL were reported in patients with MS including the early stages [2–4]. Structural changes in specific brain regions were found to be related to severity of neuropsychiatric symptoms in patients with MS. Specifically, global cortical atrophy and atrophy of the thalamus and basal ganglia were associated with increased depressive symptoms and fatigue [5–7]. Increased lesion load in frontal and temporal lobes was associated with increased depressive symptoms [8].

Since the increased neuropsychiatric symptoms and decreased HRQoL are present in the early stages of MS, one would expect that the similar changes might be present in patients with clinically isolated syndrome (CIS), who are at high risk of developing MS [9]. To date, however, only a few studies have investigated neuropsychiatric symptoms in patients with CIS and their findings are inconsistent [10–12]. Some studies reported anxiety and depressive symptoms in up to 30% of patients with CIS but other studies did not replicate these findings [11, 13]. HRQoL and its association with neuropsychiatric symptoms have not consistently been studied in patients with CIS [10, 14]. A single study explored associations between lesion load and neuropsychiatric symptoms in patients with CIS and found that increased lesion load in the right temporal lobe was related to more pronounced depressive symptoms [13]. Associations of global and regional brain atrophy with neuropsychiatric symptoms have not been studied in patients with CIS.

We aimed to evaluate neuropsychiatric symptoms (depressive symptoms, anxiety, apathy and fatigue) and HRQoL, their interrelations and structural brain correlates of neuropsychiatric symptoms in a homogeneous cohort of patients with CIS. Our first aim was to describe neuropsychiatric symptoms, HRQoL and their mutual associations in patients with CIS. Our second aim was to describe associations of neuropsychiatric symptoms and HRQoL with clinical disability and cognitive functioning. The third aim was describe a pattern of structural brain changes and their associations with neuropsychiatric symptoms.

Based on the studies of patients with MS we hypothesized that neuropsychiatric symptoms, especially depressive symptoms and anxiety would be present in patients with CIS and would be related to decreased HRQoL. We also hypothesised that cortical and subcortical atrophy and increased regional lesion load would be related to neuropsychiatric symptoms in patients with CIS.

## Methods

### Participants

67 patients with CIS were recruited at the Multiple Sclerosis Centre, Charles University, 2nd Faculty of Medicine and Motol University Hospital, Czech Republic. Patients with CIS were after the first clinical episode, had objective clinical evidence of one lesion and did not have simultaneous dissemination of lesions in space and time on routine clinical brain MRI with gadolinium and thus did not meet the criteria for MS [15]. In addition, the patients with CIS met the following criteria for eligibility for treatment with disease modifying drugs: 18–55 years of age, Expanded Disability Status Scale (EDSS) less than 3.0, 2 or more hyperintensive T2 lesions on brain MRI and 2 or more oligoclonal bands in cerebrospinal fluid [16]. Only patients with CIS on interferon-beta were recruited to get a homogeneous cohort. The first symptom was treated with 3-5g of methylprednisolone and the duration of treatment with interferon-beta was at least 1 month (median 4 months). Some patients with CIS (n = 10) were on a stable dose of selective serotonin reuptake inhibitors (SSRI) for the depressive mood developed early after the onset of CIS. One of these patients met the DSM-5 criteria for depression [17]. Administration of questionnaires and time-matched experimental brain MRI (within 4 weeks) were performed more than 30 days after administration of steroids and between 1 and 12 months from the diagnosis of CIS (median 4 months).

In addition, 46 age-, gender- and education-matched healthy participants were recruited from the Motol University Hospital staff and their relatives. They were examined by an experienced neurologist and underwent administration of questionnaires and time-matched experimental brain MRI.

Patients with CIS and healthy control participants with the history of psychiatric disorders (major depression, obsessive compulsive disorder and psychotic or schizoaffective disorders), other neurological disorders (epilepsy, the history of traumatic brain injury and the history of stroke), cardiovascular diseases and the history of alcohol or drug abuse identified on the basis of medical records and/or a detailed clinical interview with an experienced neurologist have not been included in the study.

The study was approved by the institutional ethics committee of the Motol University Hospital and was performed in accordance with the ethical standards laid down in the 1964 Declaration of Helsinki and its later amendments. Written informed consent was obtained from all participants in the study.

### Questionnaires and screening cognitive assessment

A trained psychologist administered a battery of questionnaires to evaluate participants’ cognitive functioning using Multiple Sclerosis Neuropsychological Screening Questionnaire (MSNQ), neuropsychiatric symptoms using Beck Depression Inventory (BDΙ), Beck Anxiety Inventory (BAI), Apathy Evaluation Scale (AES) and Fatigue Severity Scale (FSS), and HRQoL using Short-Form Health Survey-36 (SF-36). The MSNQ is a 15-item self-report screening measure of neuropsychological functioning with the total score range between 0 and 60, where the higher score indicates lower neuropsychological functioning [18]. The BDI is a 21-item self-report questionnaire measuring the severity of depressive symptoms with the total score range between 0 and 63, where the higher score indicates more severe depressive symptoms [19]. The BAI is a 21-item self-report questionnaire measuring the severity of anxiety with the total score range between 0 and 6, where the higher score indicates more severe anxiety [20]. The AES is an 18-item questionnaire (self-rated version) measuring the severity of apathy with the total score range between 18 and 72, where the higher score indicates more severe apathy [21]. The FSS is a 9-item self-report questionnaire measuring the severity of fatigue with the total score range between 9 and 63, where the higher score indicates more severe fatigue [22]. The SF-36 is a 36-item self-reported questionnaire of responder’s health evaluating eight health concepts: physical functioning, role limitations due to physical health problems, role limitations due to emotional problems, energy/fatigue, emotional well-being, social functioning, bodily pain and general health perception [23]. The two-step process of scoring the SF-36 includes recoding the pre-coded numeric value for each item into a scale with scores ranging between 0 and 100, where the higher score indicates the favourable health state, and averaging the items in the same scale together to create the scores of eight scales representing eight health concepts.

An experienced neurologist administered a Symbol Digit Modalities Test (SDMT) [24]. The test was administered during a clinical visit between initiation of the treatment with interferon-beta and administration of questionnaires in patients with CIS and also during a first clinical visit in control participants. The SDMT is a neuropsychological test of cognitive processing speed that has been shown to be the most sensitive measure of cognitive impairment in patients with MS strongly associated with grey matter atrophy and increased lesion load [25, 26].

### Magnetic resonance imaging acquisition and analysis

Brain MRI was performed at 1.5T device (Siemens AG, Erlangen, Germany) in an experimental protocol using 1) T1-weighted 3-dimensional high resolution magnetization-prepared rapid acquisition with gradient echo (MP-RAGE) sequence with the following parameters: TR/TE = 12/4.605 ms, flip angle 15°, 150 continuous partitions and slice thickness 1.0 mm for volumetric measurement and 2) fluid-attenuated inversion recovery (FLAIR) sequence with the following parameters: TR/TE/TI = 11000/140/2600 ms, flip angle 90°, 100 continuous partitions, and slice thickness 1.5 mm for lesion load measurement. The scans were visually inspected by an experienced neuroradiologist to ensure appropriate data quality. The experimental protocol was available for all patients with CIS and 31 control participants.

Brain tissue volume (normalized brain parenchymal [nBP], grey and white matter [nGM and nWM] volumes) was estimated with SIENAX, a part of FSL http://fsl.fmrib.ox.ac.uk/fsl/fslwiki/SIENA [27]. Voxel-based morphometry (VBM) was used to assess focal differences in brain anatomy. VBM was performed with masking of the registration cost function with lesion masks to reduce the impact of WM lesions on brain segmentation and creation of GM template. Lesions masks were obtained by Lesion Segmentation Tool (LST) toolbox version 2.0.15 (www.statistical-modelling.de/lst.html) for SPM (http://www.fil.ion.ucl.ac.uk/spm/).The algorithm segmented the T1 images into the three main tissue classes (cerebrospinal fluid, GM and WM). This information was then combined with the coregistered FLAIR intensities to calculate lesion probability maps. A lesion filling algorithm implemented in LST toolbox was adopted. The algorithm uses previously generated lesion masks registered to the image to fill the lesions with intensities matching the surrounding normal appearing WM. An optimized VBM approach was adopted with all processing steps carried out using openware FSL version 5.0.7 (http://fsl.fmrib.ox.ac.uk/fsl/fslwiki/FSLVBM). Anatomical localization of significant clusters was established using the MNI Structural Atlas. Lesion load for each brain lobe was obtained by coregistration of lesion masks to standard MNI brain using FLIRT and establishing anatomical localization of lesions using the MNI Structural Atlas [28, 29].

### Statistics

Students’ independent two-sample t-tests evaluated mean differences between the groups in age, years of education, MSNQ and SDMT scores and nBP, nWM and nGM volumes. The χ^2^ test evaluated differences in gender proportions. As the assumption of normality was breached (values of skewness and curtosis ranged outside -1 to +1) for most of the neuropsychiatric questionnaires’ and SF-36 health concept scores, we used a non-parametric (NP) Mann-Whitney U test to evaluate differences in these variables. A general linear model (GLM) implemented in FSL was used to compare voxel-wise differences in regional cortical and subcortical grey matter volumes derived from VBM. Pearson correlation coefficient was calculated to explore bivariate relationships between BDI, BAI, AES and FSS scores and each SF-36 health concept score. Holm-Bonferroni correction for multiple comparisons was used in the correlation analysis to provide more conservative estimates of the hypothesized associations. If a correlational analysis between neuropsychiatric symptoms and SF-36 health concept scores yielded a significant association, a linear regression model adjusted for age, gender, years of education and SSRI medication was estimated. Correlation coefficients for continuous variables––MSNQ and SDMT scores (Pearson) and an ordinal variable––EDSS score (Spearman) were calculated to explore their bivariate relationships with BDI, BAI, AES and FSS scores and each SF-36 health concept score. Holm-Bonferroni correction for multiple comparisons was also used. Again, if a correlational analysis yielded a significant association, a covariate-adjusted linear regression model was estimated. The relationships of nBP, nWM, nGM, regional cortical and subcortical GM volumes (derived from VBM) and total and regional lesion load volumes with BDI, BAI, AES and FSS scores were assessed using Pearson’s correlation and GLM correlation models implemented in FSL, respectively. The results of VBM were corrected for family-wise error using a FSL's tool for nonparametric permutation inference [30]. Again, if a correlational analysis between neuropsychiatric symptoms and MRI data yielded a significant association, a covariate-adjusted linear regression model was estimated. Statistical significance was set at two-tailed alpha of 0.05. Effect sizes were reported using Cohen’s d (d) for the t-test and effect-size r score (r) for the Mann-Whitney U test. With our sample size, effect-size r score of about 0.45 corresponds to Cohen’s d of 1.0. Analyses were conducted with IBM SPSS 20.0 software.

## Results

The groups did not differ in age, gender, education, and MSNQ score. The CIS group had lower SDMT score compared to the control group (p = 0.001, d = 0.64). The results are presented in Table 1.

**Table 1:** Characteristics of the study participants.

	Controls (n = 46)	CIS (n = 67)	p values
Age (years)	29.96 (8.70)	32.30 (8.22)	.144^a^
Women, n (%)	25 (54.3)	36 (53.7)	.948^b^
Education (years)	16.13 (2.80)	15.13 (3.00)	.072^a^
EDSS	NA	1.55 (0.58)	NA
MSNQ (score)	13.20 (7.53)	11.46 (7.85)	.239^a^
SDMT (score)	63.17 (11.6)	56.27 (10.35)	.001^a^
BDI (score)	4.48 (4.91)	6.61 (6.05)	.026^c^
BAI (score)	5.09 (5.32)	7.63 (6.06)	.006^c^
AES (score)	31.17 (8.47)	32.64 (7.15)	.164^c^
FSS (score)	28.39 (12.56)	29.50 (12.02)	.601^c^
SF-36 Physical functioning (score)	96.41 (6.64)	90.64 (14.62)	.003^c^
SF-36 Limitations physical (score)	84.24 (28.56)	74.30 (34.32)	.095^c^
SF-36 Limitations emotional (score)	79.59 (32.77)	82.32 (29.07)	.912^c^
SF-36 Energy/fatigue (score)	61.50 (16.37)	58.37 (17.95)	.412^c^
SF-36 Emotional well-being (score)	76.04 (16.51)	72.54 (15.15)	.156^c^
SF-36 Social functioning (score)	88.67 (15.72)	80.94 (20.83)	.037^c^
SF-36 Bodily pain (score)	90.91 (15.00)	79.93 (20.68)	.002^c^
SF-36 General health (score)	74.89 (15.04)	56.23 (21.15)	< .001^c^
nBP (volume; cm^3^)	1506.69 (56.27)^d^	1463.95 (71.92)	.004^a^
nWM (volume; cm^3^)	702.95 (35.81)^d^	682.83 (36.18)	.012^a^
mGM (volume; cm^3^)	803.74 (36.92)^d^	781.11 (53.88)	.037^a^
Lesion Load (volume; cm^3^)	N.A.	2.61 (4.05)	N.A.
Lesion Load, median (IQR) (volume; cm^3^)	N.A.	1.13 (1.98)	N.A.

The values represent mean (SD) unless indicated otherwise.

CIS: clinically isolated syndrome; EDSS: Expanded Disability Status Scale; MSNQ: Multiple Sclerosis Neuropsychological Questionnaire; SDMT: Symbol Digit Modalities Test; BDI: Beck Depression Inventory; BAI: Beck Anxiety Inventory; AES: Apathy Evaluation Scale; FSS: Fatigue Severity Scale; SF-36 Limitations physical: Role limitations due to physical health; SF-36 Limitations emotional: Role limitations due to emotional problems; nBP: normalized brain parenchymal volume; nWM: normalized white matter volume; nGM: normalized grey matter volume; IQR: interquartile range.

^a^ Students’ independent two-sample t-test.

^b^ χ^2^ test.

^c^ Mann-Whitney U test.

^d^ Based on a sample restricted to those who underwent an experimental MRI protocol (n = 31).

### HRQoL and neuropsychiatric symptoms

The CIS group had higher scores (i.e. more symptoms) in BDI and BAI (p_NP_ = 0.026, r = 0.21 and p_NP_ = 0.006, r = 0.26). Mild depressive symptoms (BDI scores between 10 and 18) were present in 15 patients with CIS and moderate depressive symptoms (BDI scores between 19 and 29) were present in 4 patients with CIS. Mild anxiety (BAI scores between 10 and 18) was present in 20 patients with CIS, moderate anxiety (BAI scores between 19 and 29) was present in 2 patients with CIS and severe anxiety (BAI scores between 30 and 63) was present in 1 patient with CIS. Among the patients with CIS on SSRI medication, 5 patients had mild depressive symptoms, 4 patients had mild anxiety and 2 patients had moderate anxiety. The patient on SSRI medication meeting the criteria for depression had mild depressive symptoms and mild anxiety. The groups were similar in AES and FSS scores. The CIS group had lower scores (i.e. more limitation/symptoms) in “physical functioning” (p_NP_ = 0.003, r = 0.28), “social functioning” (p_NP_ = 0.037, r = 0.19), “bodily pain” (p_NP_ = 0.002, r = 0.28) and “general health” (p_NP_<0.001, r = 0.44) SF-36 scales. Excluding the patient with CIS meeting the DSM-5 criteria for depression did not essentially change the results. The results are presented in Table 1.

In the CIS group, BDI, BAI and FSS scores correlated with “role limitations due to physical health”, “role limitations due to emotional problems”, “energy/fatigue”, “emotional well-being”, “bodily pain” and “general health” SF-36 scales. In addition, BDI and BAI scores correlated with “social functioning” SF-36 scale. The AES score correlated with “role limitations due to emotional problems” and “emotional well-being” SF-36 scales. The results are presented in Table 2. These associations for BDI (standardized regression coefficient [β]≤-0.34, p≤0.005), BAI (β≤-0.37, p≤0.003), AES (β≤-0.46, p<0.001) and FAS (β≤-0.37, p≤0.003) scores remained significant in the regression analyses adjusted for age, gender, education and SSRI medication.

**Table 2:** Association of neuropsychiatric symptoms with health-related quality of life in clinically isolated syndrome.

	BDI	BAI	AES	FSS
SF-36 Physical functioning (score)	-0.126	-0.152	-0.144	-0.130
SF-36 Limitations physical (score)	-0.374**	-0.362**	0.075	-0.389**
SF-36 Limitations emotional (score)	-0.511***	-0.455***	-0.445***	-0.383**
SF-36 Energy/fatigue (score)	-0.472***	-0.499***	-0.300*	-0.530***
SF-36 Emotional well-being (score)	-0.560***	-0.609***	-0.556***	-0.477***
SF-36 Social functioning (score)	-0.499***	-0.523***	-0.125	-0.220
SF-36 Bodily pain (score)	-0.335**	-0.381**	-0.164	-0.477***
SF-36 General health (score)	-0.367**	-0.412***	-0.207	-0.399**

* p < .05.

** p < .01.

*** p < .001.

Values in bold indicate significant correlations after Holm-Bonferroni correction for multiple comparisons.

BDI: Beck Depression Inventory; BAI: Beck Anxiety Inventory; AES: Apathy Evaluation Scale; FSS: Fatigue Severity Scale; SF-36 Limitations physical: Role limitations due to physical health; SF-36 Limitations emotional: Role limitations due to emotional problems.

### Associations of EDSS, MSNQ and SDMT scores with neuropsychiatric symptoms and HRQoL

There was no correlation of the EDSS score with neuropsychiatric symptoms and any SF-36 scale scores. The MSNQ score correlated with BDI and BAI scores and with “role limitations due to emotional problems”, “energy/fatigue” and “emotional well-being” SF-36 scales in the CIS group. The SDMT score correlated with the BDI score and with “energy/fatigue”, “emotional well-being” and “bodily pain” SF-36 scales in the CIS group. The results are presented in Table 3. These associations remained significant in the covariate-adjusted regression models (β≤-0.29, p≤0.019).

**Table 3:** Association of EDSS, MSNQ and SDMT scores with neuropsychiatric symptoms and health-related quality of life in clinically isolated syndrome.

	EDSS	MSNQ	SDMT
BDI (score)	0.085	0.563**	-0.345**
BAI (score)	0.126	0.477**	-0.283*
AES (score)	0.220	0.257*	-0.228
FSS (score)	-0.006	0.290*	-0.146
SF-36 Physical functioning (score)	-0.241*	0.084	0.226
SF-36 Limitations physical (score)	-0.137	-0.224	0.166
SF-36 Limitations emotional (score)	0.012	-0.442**	0.247*
SF-36 Energy/fatigue (score)	-0.046	-0.355**	0.330**
SF-36 Emotional well-being (score)	-0.113	-0.506**	0.411**
SF-36 Social functioning (score)	-0.100	-0.236*	0.195
SF-36 Bodily pain (score)	-0.025	-0.273*	0.321**
SF-36 General health (score)	-0.137	-0.225	0.281*

* p < .05.

** p < .01.

Values in bold indicate significant correlations after Holm-Bonferroni correction for multiple comparisons.

EDSS: Expanded Disability Status Scale; MSNQ: Multiple Sclerosis Neuropsychological Questionnaire; SDMT: Symbol Digit Modalities Test; BDI: Beck Depression Inventory; BAI: Beck Anxiety Inventory; AES: Apathy Evaluation Scale; FSS: Fatigue Severity Scale; SF-36 Limitations physical: Role limitations due to physical health; SF-36 Limitations emotional: Role limitations due to emotional problems.

### Associations of structural brain changes with neuropsychiatric symptoms

**Fig 1:**
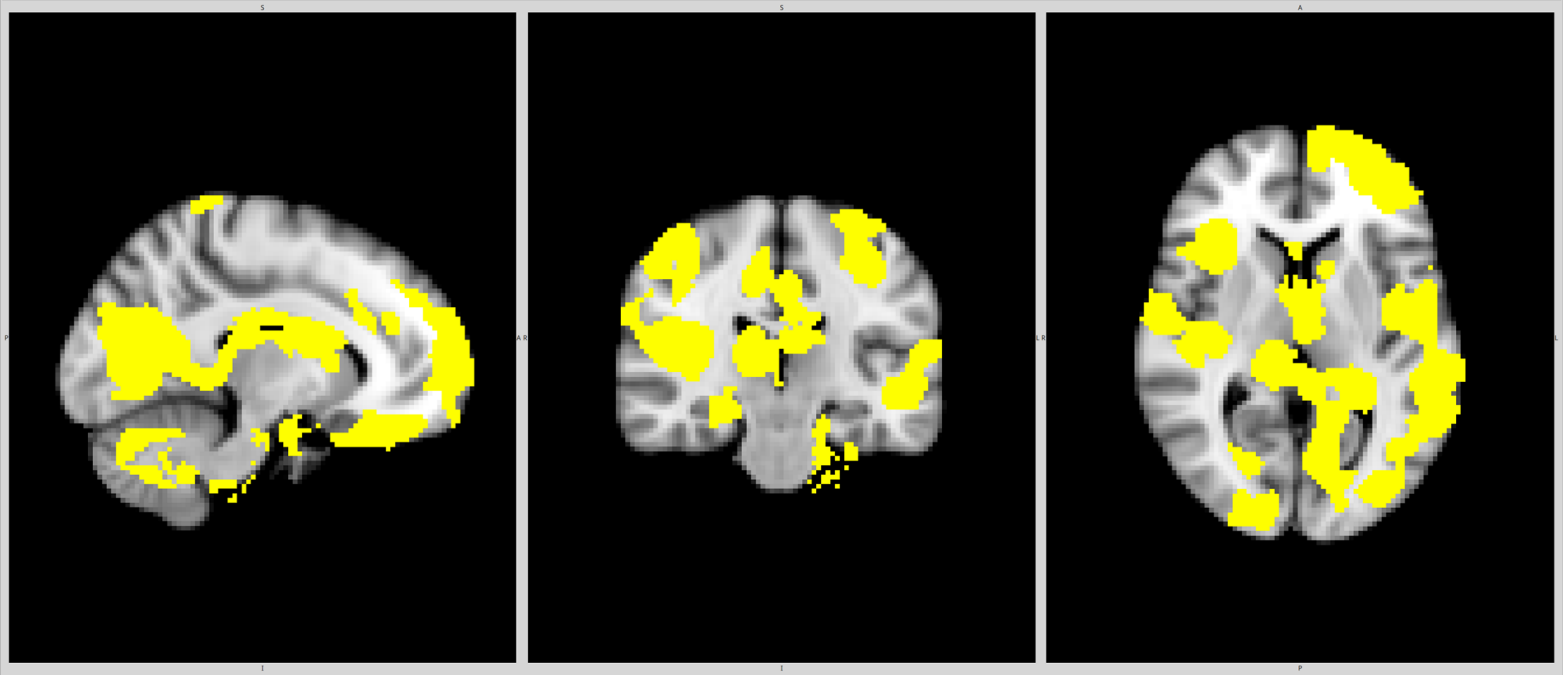
Regional brain volumes reduced in clinically isolated syndrome compared to controls.

**Fig 2:**
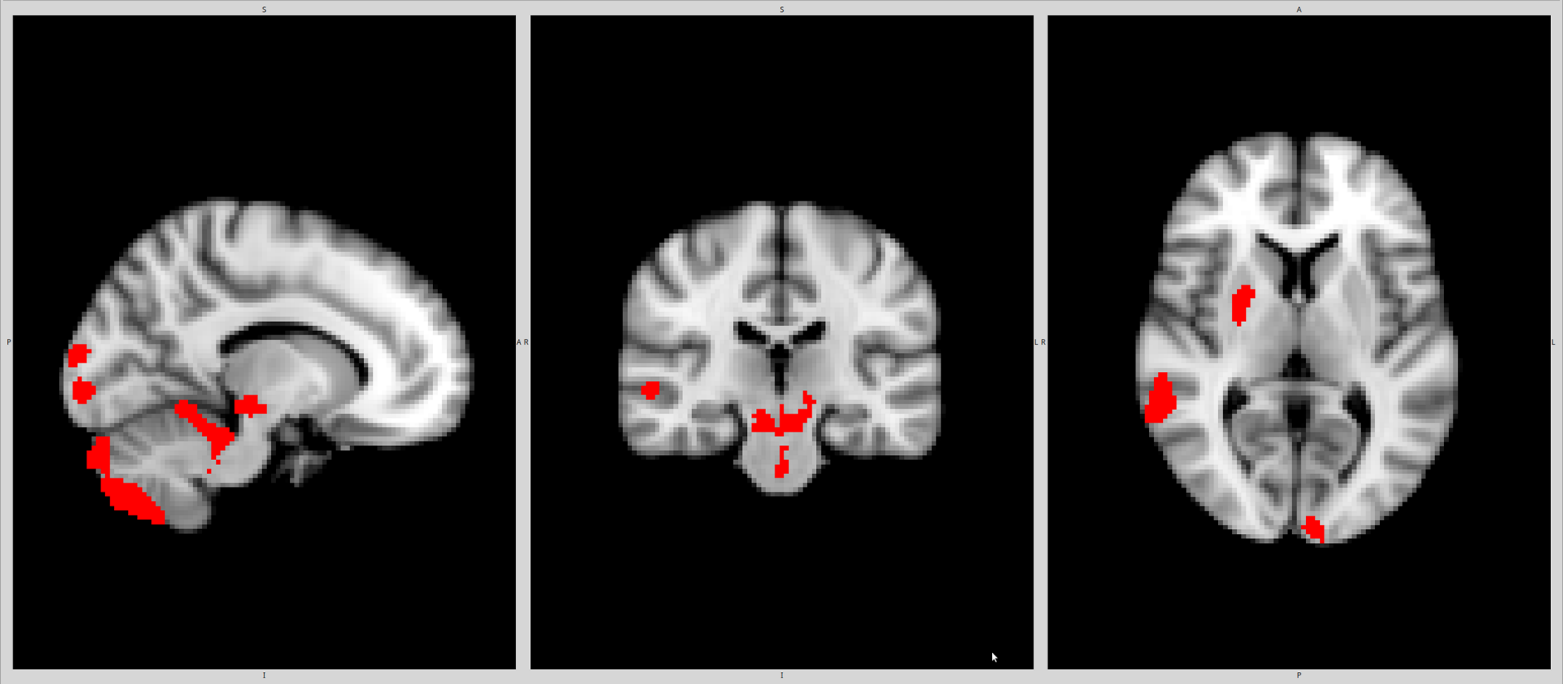
Regional brain volumes increased in clinically isolated syndrome compared to controls.

The CIS group had reduced nBP (p = 0.004, d = 0.60), nWM (p = 0.012, d = 0.52), nGM (p = 0.037, d = 0.43), cortical (p≤0.006), thalamic, caudate, right putamen and cerebellar volumes (p<0.001). The FSL-VBM results are presented in S1 and S2 Tables and Figs 1 and 2. Total and regional lesion load is presented in Table 1 and S3 Table. Higher BAI scores correlated with lower nWM volume (r = -0.27, p = 0.030) in the CIS group. Higher BDI scores correlated with higher lesion load in the right temporal lobe (r = 0.30, p = 0.013). Higher AES scores correlated with higher lesion load in the right and left insulas and right occipital lobe (r≥0.29, p≤0.026). These associations except for the association between the AES score and right insular lesion load remained significant in the covariate-adjusted regression models (BAI: ß = -0.25, p = 0.045; BDI: ß = 0.32, p = 0.010 and AES: ß≥0.29, p≤0.032). Other associations between neuropsychiatric symptoms and MRI data were not significant.

## Discussion

We evaluated neuropsychiatric symptoms in a homogeneous cohort of patients with CIS and using BDI and BAI questionnaires we demonstrated more depressive symptoms and anxiety at the group level in these individuals compared to demographically-matched healthy controls. Depressive symptoms and anxiety were found in 28% and 34%, respectively, of patients with CIS and the symptoms were mostly mild. In patients with depressive symptoms and anxiety, 26% of them were on SSRI medication and 1 patient met the DSM-5 criteria for depression. Similarly, a longitudinal study reported anxiety and depressive symptoms in 30% of patients with CIS at baseline where a higher occurrence of depression at follow-up was associated with clinical progression [13]. These findings have not consistently been replicated in other studies, which were, however, underpowered due to the small sample sizes (n≤18) [10, 11]. It should be noted that the mean BDI and BAI scores of CIS patients were within the normal range in our study. Apathy has not been studied in patients with CIS and our results indicated that apathy may not be a common feature of patients with CIS, unlike in patients with MS [31]. A single study evaluated fatigue in patients with CIS and found that fatigue is an independent predictor of conversion to clinically definite MS [32]. Our results do not support the notion that fatigue is present in patients with CIS. Different findings between the present and the previous study may be explained by the fact that fatigue is related to disease duration and patients in the present study were examined shortly after diagnosis of CIS, before fatigue could develop [33].

The HRQoL is decreased in patients with MS and this applies to all eight health concepts (scores) of the SF-36 questionnaire [2, 34]. Decreased HRQoL in mental (emotional and social) and physical scores of the SF-36 questionnaire was found to be associated with neuropsychiatric symptoms in patients with MS [2, 4, 6, 35]. In patients with CIS, a single study with a small sample size (n = 18) indicated reduced HRQoL, but did not found any association of HRQoL with depressive symptoms and anxiety [10]. Our findings indicated that HRQoL is decreased, specifically in physical and social functioning, in patients with CIS. In patients with CIS, increased depressive symptoms and anxiety were related to decrease of almost all HRQoL scores of the SF-36 questionnaire, which is a similar finding to that of patients with MS [2, 4]. Increased apathy and fatigue were related to decrease of emotional scores and almost all HRQoL scores of the SF-36 questionnaire, respectively. Even though apathy and fatigue have not been increased in patients with CIS, it seems that they may have a negative impact on their HRQoL similarly to that of patients with MS [2].

In patients with MS, increased neuropsychiatric symptoms and decreased HRQoL have been related to more pronounced clinical disability and cognitive dysfunction since the early stages and this relation was stronger with increasing severity of the disease [11, 18, 35, 36]. In patients with CIS, a single study found association between self-perceived cognitive functioning and increased depressive symptoms, but did not study association with HRQoL [37]. Associations of clinical disability with neuropsychiatric symptoms and HRQoL have not been studied in patients with CIS. Our findings indicate that slower cognitive processing speed and self-perceived lower cognitive functioning are associated with higher depression scores and decreased HRQoL emotional scores of the SF-36 questionnaire in patients with CIS. Similar findings of association between cognitive processing speed, self-reported cognitive functioning and emotional changes were reported previously in patients with MS [18, 38]. In this study on patients with CIS, clinical disability was not associated with neuropsychiatric symptoms and HRQoL, which may be explained by very mild clinical disability (mean EDSS 1.55) in these individuals.

The aetiology of neuropsychiatric symptoms in patients with MS is multifactorial and seems to represent a combination of psychological reaction to a chronic and potentially invalidating disease and structural brain changes including increased lesion load in and atrophy of frontal, temporal and parietal grey and white matter [1, 2, 6, 8, 39]. In patients with CIS, a single study found association between increased lesion load in the right temporal lobe and more pronounced depressive symptoms [13]. In the present study, patients with CIS had widespread global and regional cortical atrophy, predominantly in frontal and temporal lobes, together with subcortical atrophy, predominantly in the thalamus, which corresponds with previous findings [40, 41]. Using the BDI, AES and BAI scales we found that increased depressive symptoms, apathy and anxiety were associated with higher lesion load in the right temporal lobe, left insula and right occipital lobe, and WM atrophy, respectively, above and beyond age, gender, education and SSRI medication. These findings thus indicate that neuropsychiatric symptoms in addition to psychological reaction to the illness reflect also structural brain changes in specific brain regions similarly to that found in patients with MS [1, 39].

One of the strengths of this study is the fact that we used a homogeneous sample of patients with CIS, characterized their four core neuropsychiatric symptoms and HRQoL using relevant questionnaires and measured in detail their structural brain changes including regional brain atrophy and regional lesion load. In addition, this is the first study to evaluate eight health concepts of HRQoL and apathy in patients with CIS. This study also has limitations. This was a cross-sectional study, which does not allow for evaluating the predictive value of neuropsychiatric symptoms for conversion to clinically definite MS and tracking their changes over time. The results may be influenced by treatment with interferon-beta, which may affect neuropsychiatric symptoms in both positive and negative ways [42, 43]. For identification of psychiatric disorders in the participants we used the unstructured clinical interview and medical records unlike the structured diagnostic interview, which would determine whether the patients with CIS may fulfil criteria for mental health disorders. Cognitive functioning was evaluated by a self-report questionnaire and a single neuropsychological test, which may not properly reflect the general cognitive status [37]. Experimental brain MRI was available only in a subset of healthy control participants. Because of these limitations our results should be interpreted with caution. Finally, we were not able to explore the changes in HRQoL among the patients with CIS meeting the criteria for mood and anxiety disorders. Further studies are required to resolve whether those patients have associated differences in HRQoL.

In conclusion, we demonstrated increased, mostly mild, depressive symptoms and anxiety, decreased HRQoL and a negative association between neuropsychiatric symptoms and HRQoL in patients with CIS. Our findings indicate that neuropsychiatric symptoms contributing to decreased HRQoL may not be only a psychological reaction to unfavourable diagnosis but also a result of disease activity itself and thus complex therapeutic approach including antidepressant therapy, disease modifying drugs and psychosocial intervention may be beneficial in patients with CIS.

## Supporting information

S1 Table Regional brain volumes reduced in clinically isolated syndrome compared to controls. (DOCX)

S2 Table Regional brain volumes increased in clinically isolated syndrome compared to controls. (DOCX)

S3 Table Regional lesion load volumes in clinically isolated syndrome. (DOCX)
