# Supplementary material for: Health-related quality of life, neuropsychiatric symptoms and structural brain changes in clinically isolated syndrome
Source: PLoS One. 2018 Jul 6;13(7):e0200254. doi: 10.1371/journal.pone.0200254 (PMC6034869; doi:10.1371/journal.pone.0200254)
Supplement: S1 Table — (DOCX) [file pone.0200254.s001.docx]

**Supplementary Table 1. Regional brain volumes reduced in clinically isolated syndrome compared to controls.**

| Structure | Side | Volume (voxels) | Volume (mm^3­­^) | maxX (mm) | maxY (mm) | maxZ (mm) | P value |
| --- | --- | --- | --- | --- | --- | --- | --- |
| Frontal Lobe | lh | 8861 | 70888 | -4 | 34 | -32 | <0.001 |
|  | rh | 5836 | 46688 | 18 | 14 | -32 | <0.001 |
| Temporal Lobe | lh | 6765 | 54120 | -28 | -6 | -54 | <0.001 |
|  | rh | 3855 | 30840 | 32 | 16 | -48 | <0.001 |
| Insula | lh | 459 | 3672 | -36 | -4 | -12 | <0.001 |
|  | rh | 979 | 7832 | 30 | 16 | -16 | 0.006 |
| Parietal Lobe | lh | 4674 | 37392 | -12 | -46 | -2 | <0.001 |
|  | rh | 4184 | 33472 | 22 | -38 | -8 | <0.001 |
| Occipital Lobe | lh | 3108 | 24864 | -8 | -76 | -8 | <0.001 |
|  | rh | 2013 | 16104 | 22 | -40 | -16 | <0.001 |
| Periventricular white matter | lh | 1075 | 8600 | 0 | 12 | -4 | <0.001 |
|  | rh | 681 | 5448 | 2 | 10 | -10 | <0.001 |
| Caudate | lh | 526 | 4208 | -2 | 10 | -8 | <0.001 |
|  | rh | 275 | 2200 | 12 | 16 | -12 | <0.001 |
| Putamen | rh | 79 | 632 | 26 | 20 | -10 | <0.001 |
| Thalamus | lh | 875 | 7000 | 0 | -12 | -8 | <0.001 |
|  | rh | 795 | 6360 | 2 | -18 | -8 | <0.001 |
| Cerebellum | lh | 2431 | 19448 | -10 | -36 | -52 | <0.001 |
|  | rh | 4444 | 35552 | 32 | -56 | -64 | <0.001 |
