# Supplementary material for: Health-related quality of life, neuropsychiatric symptoms and structural brain changes in clinically isolated syndrome
Source: PLoS One. 2018 Jul 6;13(7):e0200254. doi: 10.1371/journal.pone.0200254 (PMC6034869; doi:10.1371/journal.pone.0200254)
Supplement: S2 Table — (DOCX) [file pone.0200254.s002.docx]

**Supplementary Table 2. Regional brain volumes increased in clinically isolated syndrome compared to controls.**

| Structure | Side | Volume (voxels) | Volume (mm^3­­^) | maxX (mm) | maxY (mm) | maxZ (mm) | P value |
| --- | --- | --- | --- | --- | --- | --- | --- |
| Temporal Lobe | lh | 94 | 752 | -36 | -76 | -22 | <0.001 |
|  | rh | 603 | 4824 | 30 | -4 | -12 | 0.002 |
| Insula | rh | 94 | 752 | 44 | 6 | -4 | 0.008 |
| Occipital Lobe | lh | 1247 | 9976 | -32 | -82 | -24 | <0.001 |
|  | rh | 358 | 2864 | 20 | -94 | -26 | 0.003 |
| Periventricular white matter | lh | 41 | 328 | -8 | -32 | -18 | <0.001 |
| Putamen | rh | 542 | 4336 | 32 | -6 | 48 | 0.002 |
| Cerebellum | lh | 3792 | 30336 | -24 | -66 | -64 | <0.001 |
|  | rh | 1841 | 14728 | 2 | -44 | -54 | <0.001 |
