# Supplementary material for: Health-related quality of life, neuropsychiatric symptoms and structural brain changes in clinically isolated syndrome
Source: PLoS One. 2018 Jul 6;13(7):e0200254. doi: 10.1371/journal.pone.0200254 (PMC6034869; doi:10.1371/journal.pone.0200254)
Supplement: S3 Table — (DOCX) [file pone.0200254.s003.docx]

**Supplementary Table 3. Regional lesion load volumes in clinically isolated syndrome.**

| Region | Left-sided lesion load (volume; mm^3^) | Right-sided lesion load (volume; mm^3^) |
| --- | --- | --- |
| Frontal lobe | 76.17 (193.19) | 81.21 (172.59) |
| Temporal lobe | 113.52 (293.73) | 117.34 (237.07) |
| Insula | 2.09 (7.44) | 11.44 (75.92) |
| Parietal lobe | 144.13 (299.30) | 204.28 (492.82) |
| Occipital lobe | 205.24 (374.87) | 136.44 (226.72) |
| Periventricular white matter | 573.52 (1102.93) | 751.44 (1362.89) |
| Deep grey matter | 103.63 (173.69) | 85.19 (150.27) |
| Cerebellum | 3.45 (16.36) | 1.96 (8.58) |

The values represent mean (SD).
